# Childhood emotional trauma and social avoidance and distress in adolescents: psychological resilience as mediator and left-behind experience as moderator

**DOI:** 10.3389/fpsyg.2025.1578809

**Published:** 2025-07-14

**Authors:** Ding Zhang, Nan Gao, Xuelan Liu, Yao Wang

**Affiliations:** ^1^School of Education, South China Normal University, Guangzhou, China; ^2^Center of Student Psychological Development, Zhoukou Normal University, Zhoukou, China; ^3^School of Ethnology and Sociology, South-Central University for Nationalities, Wuhan, China; ^4^South China Normal University of Psychology, Guangzhou, China

**Keywords:** childhood emotional trauma, social avoidance and distress, psychological resilience, left-behind experience, adolescents

## Abstract

**Introduction:**

Adolescents are navigating a critical phase of developmental transition, characterized by a profound need for interpersonal communication and mutual understanding. This study investigates the relationship between childhood emotional trauma and social avoidance and distress among junior high school students, with the goal of establishing effective interpersonal interaction patterns and fostering the healthy physical and mental development of adolescents.

**Methods:**

A model was developed to examine the mediating role of psychological resilience in the relationship between childhood emotional trauma and social avoidance and distress, as well as the moderating role of left-behind experience within this mediating pathway. Data from 577 students were analyzed using SPSS 22.

**Results:**

(1) Childhood emotional trauma significantly and positively predicts social avoidance and distress; (2) psychological resilience mediates the relationship between childhood emotional trauma and social avoidance and distress; (3) left-behind experience moderates the association between psychological resilience and social avoidance and distress.

**Discussion:**

These findings contribute to the theoretical understanding of childhood trauma and its social consequences, while offering practical insights for addressing social avoidance and distress among junior high school students. The study also discusses its theoretical and practical implications, limitations, and potential directions for future research.

## 1 Introduction

Social avoidance and distress refer to a state of continuous anxiety about social scenes, which leads to individuals not daring to communicate with others, resulting in emotional reactions of distress (Watson and Friend, 1969). Junior high school students are in a critical period of emotional instability and relationship building, and social problems tend to occur during this period (Sumter et al., 2009). If early symptoms are not effectively intervened, in turn, more serious social problems can develop (Nordmann and Herbert, 1996). Studies have shown that chronic social avoidance can lead to anxiety, depression, a serious decline in the quality of social relationships, and even addiction disorders or suicidal problems (Clifford et al., 2021; Wu et al., 2019). Therefore, it is important to explore the risk factors and occurrence mechanisms of social avoidance and distress in junior high school students for early prevention and effective intervention.

The family is the first environment in which a child grows up and the foundation on which parent–child relationships are built and developed. The risk factors experienced in childhood will have a profound impact on their healthy physical and mental development. Research shows that childhood emotional trauma affects up to 48% of the general population (Goldsmith et al., 2013) and is associated with outcomes of borderline personality disorder (BPD; Porter et al., 2020), depression (Xu et al., 2023), and anxiety (Martin-Gagnon et al., 2023) in adolescents. Emotional trauma can arise from both isolated incidents and the long-term negative impact of parents or caregivers failing to provide a supportive and developmentally appropriate environment for the child, such as through blaming, threatening, discriminating against, and other mental rejection or hostile treatment (Hart and Brassard, 1991). In previous studies, the results of childhood emotional trauma and adolescent social problems were not completely consistent. Butchart et al.’s (2006) study showed that children who experienced childhood emotional trauma were prone to social problems. Another study lacked evidence to show that childhood emotional trauma did not have a complete effect on social problems (Eun-Seok and Yon, 2018). Therefore, in light of the inconsistent studies above, there is a need to clarify the pattern of the relationship.

Exploring the potential mechanisms between childhood emotional trauma and social avoidance and distress in middle school students is also an important question for this study. Previous studies have elucidated the mediating role of negative social self-concept (Eun-Seok and Yon, 2018) and navigating social support (Guo and Huang, 2022) in the relationship. According to the process model of psychological resilience, psychological resilience is not static. Various protective and risk factors, both internal and external to the individual, can work together to influence psychological resilience, which may ultimately lead to increased, restored, or decreased levels of psychological resilience (Richardson, 2002). Connor and Davidson (2003) have also demonstrated that psychological resilience is variable and can be enhanced through intervention. Therefore, it is important to explore the relationship between psychological resilience and childhood emotional trauma, social avoidance, and distress from the perspective of mitigating the impact of negative childhood life experiences on mental health.

Left-behind experience refers to the situation where one or both parents go out to work and are away, unable to stay with the child for more than 1 year (Yu et al., 2022). Numerous studies have shown varying degrees of psychological and behavioral problems among secondary students with such experiences (Wang and Jia, 2020). For junior high school students with left-behind experiences, this situation increases their risk of neglect within the family and makes them more vulnerable to psychological problems. However, on the other hand, the left-behind experience may also empower individuals to acquire the skills of independent living, coupled with a high level of resilience to cope with frustration, which can buffer the effects of childhood maltreatment (Zhou et al., 2019). Previous studies have primarily explored the differences between left-behind and non-left-behind individuals in terms of childhood trauma, psychological resilience, social avoidance, and distress as isolated variables without examining the complexities of their interrelationships (Zhang and Xu, 2022; Wu et al., 2021). Therefore, it is imperative to delve deeper into the moderating role of left-behind experience.

To sum up, there remains a significant gap in our understanding of the impact mechanisms of childhood emotional trauma, social avoidance, and distress among Chinese middle school students. The aim of this study is to investigate the influence of childhood emotional trauma on social avoidance among junior high school students, the mediating role of psychological resilience, and the moderating role of left-behind experience, focusing on adolescents aged 12–17 years. This exploration holds significant implications for reducing the levels of social avoidance and distress experienced by these students.

### 1.1 Childhood emotional trauma and social avoidance and distress

Childhood emotional abuse is a persistent pattern of interactions between children and their abusers, potentially more detrimental to children’s psychological development than other forms of abuse. Its negative effects can persist into adulthood (Dye, 2020; van Vugt et al., 2014). Research has shown that adolescents who have experienced emotional trauma in childhood are unable to establish healthy interactions with their caregivers. Later in life, they often display difficulties in social adjustment, social anxiety, social withdrawal, and even social disorders in their interactions. Additionally, they exhibit higher levels of maladaptive emotional symptoms, such as anger, loneliness, and depression (Sonja et al., 2002). The dual-risk model of qualitative stress theory suggests that an individual’s personal qualities interact with external factors, and individuals with weaker qualities are more likely to create problems in their environment and respond negatively to environmental stressors (Belsky and Pluess, 2009). Early maladjustment may continue to have a negative impact on their cognition and mood throughout their lives through a continuous mechanism (Zeng et al., 2018). Based on these, we have formulated our first hypothesis:

*Hypothesis 1*: Childhood emotional trauma can significantly positively predict social avoidance and distress among junior high school students.

### 1.2 The mediating role of psychological resilience

Psychological resilience refers to an individual’s ability to adapt well in the face of life adversity, trauma, tragedy, threats, or other major life stressors. It implies a kind of “bouncing back” in the face of life stresses and setbacks (Henley, 2010). The mechanisms through which childhood trauma affects social anxiety have been explored from various perspectives, and factors such as general self-efficacy, coping styles (Xia et al., 2020), and early maladaptive schemas (Cui et al., 2011) have been found to play a role in the relationship between childhood trauma and anxious traits and emotions. Psychological resilience is an important protective factor for individuals facing complex life situations. Does it play a pivotal role in the relationship between childhood emotional trauma and social avoidance and distress among middle school students? Research has shown that early adverse experiences are negatively associated with psychological resilience (Wingo et al., 2010). In a 2010 cross-sectional survey of African Americans, it was found that emotional trauma in childhood significantly predicted their level of psychological resilience (Edwards et al., 2014), indicating that childhood emotional trauma negatively impacts the development of psychological resilience. Meanwhile, individuals with low levels of psychological resilience tend to have increased levels of social avoidance and distress (Zeng et al., 2017), suggesting that psychological resilience may play a crucial role in the relationship between emotional trauma and social avoidance and distress in adolescents. Based on these, we established our second hypothesis:

*Hypothesis 2*: Psychological resilience mediates the relationship between childhood emotional trauma and social avoidance and distress.

### 1.3 The moderating role of left-behind experience

Left-behind experience is a complex trauma and serves as an important factor affecting junior high school students’ social interaction. A large number of studies have confirmed that junior high school students with left-behind experiences suffer from varying degrees of psychological problems. Compared with individuals without such an experience, those with left-behind experience display significantly lower psychological resilience (Ling, 2022). Lacking parental affection, they are more prone to exhibiting negative emotions when confronted with pressure and difficulties, and they also experience significantly higher levels of adverse psychosomatic symptoms, such as social avoidance and distress, than those without left-behind experience (Wang D., 2022). Previous studies have established a link between left-behind experience and psychological resilience, social avoidance, and distress. Junior high school students with left-behind experience face unfavorable situations, including the absence of parental care, a cold family atmosphere, poor supervision, and limited family coping abilities in their early stages of development (Fan, 2017). These negative family environments may decrease their level of psychological resilience, weakening the protective role of psychological resilience against internalizing problems (van Vugt et al., 2014). On the other hand, junior high school students without left-behind experience tend to enjoy a better living environment and receive more psychosocial support. They are more likely to respond to social situations in a positive and optimistic manner (Wu et al., 2021). Furthermore, relevant research also indicates that left-behind experience serves as an important moderating variable in the relationship between psychological resources and other individual adaptation issues (Wu et al., 2021; Li, 2016). Based on these, we have established our third hypothesis:

*Hypothesis 3*: Left-behind experience plays a moderating role in the second half of the mediating path.

**Figure 1:**
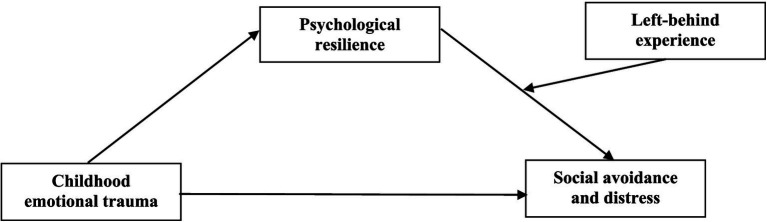
Research hypothesis model.

In summary, this study constructed a model (Figure 1) to explore the mediating and moderating mechanisms of childhood emotional trauma in predicting social avoidance and distress, aiming to provide ideas for improving the psychological resilience of junior high school students and reducing the adverse effects of emotional trauma in childhood. Three hypotheses were put forward: (1) childhood emotional trauma can significantly positively predict social avoidance and distress among junior high school students; (2) psychological resilience plays a mediating role in the relationship between childhood emotional trauma and social avoidance and distress; and (3) left-behind experience plays a moderating role in the second half of the mediating path, specifically in the relationship between psychological resilience and social avoidance and distress.

## 2 Materials and methods

### 2.1 Participants

A total of 800 students from two junior high schools in Henan Province, China, were selected using cluster sampling. After excluding questionnaires with excessively regular or missing answers, 577 valid questionnaires were collected, resulting in an effective rate of 72.1%. The sample included 174 seventh grade students, 92 eighth grade students, and 311 ninth grade students. Of the valid sample, 308 were boys and 269 were girls. There were 18 only children, while 559 were not only children. Twelve individuals came from urban areas, and 565 came from rural areas. Ninety-seven were student cadres, and 480 were not. A total of 505 fathers had high school education or below, while 72 had education above high school. Similarly, 514 mothers had a high school education or below, and 63 had an education above high school. Three hundred and four individuals had left-behind experience (i.e., one or both parents left them for more than a year before age 16), while 273 did not. The subjects ranged in age from 12 to 17 years, with a mean age of 14.45 years (SD = 0.94).

### 2.2 Variables and measures

#### 2.2.1 Childhood trauma questionnaire

The childhood trauma questionnaire compiled by Bernstein et al. (2003) and translated and revised by Zhao (2005) is the main tool for measuring childhood trauma at present, and the subjects are required to fill out the questionnaire according to their growth experience before the age of 16. There are 28 questions in the questionnaire, including five dimensions: emotional trauma, emotional neglect, physical trauma, physical neglect, and sexual trauma. The higher the score, the higher the trauma level. This study calculated the total score of emotional trauma and emotional neglect to measure the degree of emotional trauma among junior high school students. In some studies of Chinese samples, this calculation method has good reliability and validity (Zhang, 2022; Wang Z. J., 2022). In this study, the Cronbach’s *α* coefficient of the scale was 0.76. This shows that the measures are reliable.

#### 2.2.2 Social avoidance and distress questionnaire

The social avoidance and distress questionnaire, revised by Wang (1999), is adopted. The questionnaire contains 28 items, of which 14 are used to evaluate social avoidance and 14 are used to evaluate social distress. The method of “yes, no” scoring is adopted, with “yes” getting 1 point and “no” getting 0 points. The higher the score, the more serious the individual’s social avoidance and distress are. The scale has good reliability and validity in the Chinese sample (Ma et al., 2020). In this study, the Cronbach’s *α* coefficients of social avoidance and social distress were 0.75 and 0.76. This shows that the measures are reliable.

#### 2.2.3 Adolescent psychological resilience questionnaire

The adolescent psychological resilience scale compiled by Hu and Gan (2008) was adopted. There are 27 items in total, of which 12 are scored reversely, divided into two subscales of human power and support. Personal power includes three factors: goal concentration, emotional control, and positive cognition, and support includes two factors: family support and interpersonal assistance. Five points are scored (from completely inconsistent to fully consistent). The higher the score, the higher the level of psychological resilience. In this study, the Cronbach’s α coefficient of the scale was 0.78. This shows that the measures are reliable.

### 2.3 Control variables

According to previous studies, adolescents of different genders and ages have different levels of social avoidance and distress in the context of school education (Luo, 2022; Young, 2008). Therefore, in this study, gender and age were used as control variables.

### 2.4 Statistical treatment

After obtaining the informed consent of the school and the students, a written test was used in the class, and the instructions and confidentiality commitment were read by the trained instructor. SPSS version 22.0 (IBM, NY, United States) was used for the statistical analysis. Descriptive statistics were produced for all variables, while the PROCESS macro for SPSS (Model 4) was applied to examine the mediating effect of fear of negation (Hayes, 2013). The significance of the regression coefficient was tested by the bootstrap method. The sample distribution constructs 5,000 samples through playback random sampling and obtains the standard error and confidence interval of parameter estimation. In the current study, missing data were handled via maximum likelihood estimates (ML).

### 2.5 Common method deviation control

As all the survey data were derived from adolescent self-reports, there may be a potential for common method deviation. Therefore, the Harman single-factor test was employed to assess the potential common method bias among the variables. The results revealed that the eigenvalues of 15 factors exceeded 1, while the explanatory power of the first factor fell below 40% of the critical value (the value of variation was 12.46%). Consequently, it was determined that common method bias did not significantly influence the data results. SPSS 25 was utilized to conduct a descriptive statistical analysis of each measurement scale (Table 1). The standard deviations of the measurement items pertaining to childhood emotional trauma, psychological resilience, social avoidance, and distress remained relatively stable. Additionally, the skewness and kurtosis values exhibited relative stability, with all absolute values falling below 3, suggesting that the distribution of scores among the surveyed individuals is approximately normal and stable. To mitigate potential multicollinearity issues, we conducted diagnostic tests examining the correlation between predictor variables and their interaction terms. The results indicated a correlation coefficient of 0.073 between the predictor and interaction term, which is substantially below the conventional threshold of 0.7, this confirms the absence of significant multicollinearity in our analytical model.

**Table 1:** Descriptive statistics.

Variable	Skewness		Kurtosis	
	Statistic	Std. error	Statistic	Std. error
Childhood emotional trauma	1.223	0.102	1.204	0.203
Psychological resilience	0.355	0.102	0.243	0.203
Social avoidance and distress	0.004	0.102	−0.498	0.203

## 3 Results

### 3.1 The mediating effect of psychological resilience

The correlation analysis of childhood emotional trauma, psychological resilience, and social avoidance and distress showed that childhood emotional trauma was significantly positively correlated with social avoidance and distress and negatively correlated with psychological resilience, while psychological resilience was significantly negatively correlated with social avoidance and distress (Table 2).

**Table 2:** Mean, standard deviation, and correlation coefficient for each variable.

Variable	M	SD	1	2	3	4	5	6
Sex	0.47	0.50	−					
Age	14.45	0.94	0.112^**^	−				
Childhood emotional trauma	16.25	5.71	−0.005	−0.010	−			
Psychological resilience	90.01	13.17	−0.007	0.059	−0.297^**^	−		
Social avoidance and distress	16.18	6.16	−0.157^**^	−0.059	0.189^**^	−0.313^**^	−	

*N* = 577. The bootstrap method was used for calculating the correlation coefficients. Gender is a dummy variable coded 1 for girls and 0 for boys, and the mean represents the proportion of girls. ^**^p < 0.01.

According to the method suggested by Hayes (2013), this study found that childhood emotional trauma can predict social avoidance and distress and mediate psychological resilience. As Table 3 shows, in Equation 1, with social avoidance and distress as the dependent variable, childhood emotional trauma has a significant positive predictive effect on social avoidance and distress (*c_1_* = 0.185, *t* = 4.565, *p* < 0.001), and its total effect is significant. In Equation 2, with psychological resilience as the dependent variable, childhood emotional trauma had a negative predictive effect on psychological resilience (*a_1_* = −0.288, *t* = −7.206, *p* < 0.001). In Equation 3, with social avoidance and distress as the dependent variable, psychological resilience had a significant effect on social avoidance and distress (*b_1_* = −0.281, *t* = −6.905, *p* < 0.001), and childhood emotional trauma still significantly predicted the social avoidance and distress (*c’_1_* = 0.103, *t* = 2.542, *p* < 0.05). This indicates that psychological resilience partially mediates the relationship between childhood emotional trauma and social avoidance and distress. In addition, the interaction between psychological resilience and left-behind experience had a significant impact on social avoidance and distress (*b_2_* = 0.174, *t* = 2.123, *p* < 0.05), signifying that left-behind experience played moderating effect in the latter half of the mediation path. *a_1_* and *b_2_* were significant at the same time, suggesting that the moderated mediation model was established and the research hypothesis was supported.

**Table 3:** Test of moderated mediating model of childhood emotional trauma on social avoidance and distress.

	Equation 1 (social avoidance and distress)			Equation 2 (dependent variable: psychological resilience)			Equation 3 (social avoidance and distress)		
Variable	*b*	*t*	95% CI	*b*	*t*	95% CI	*b*	*t*	95% CI
Sex	−0.342	−4.206^***^	[−0.502, −0.182]	−0.027	−0.331	[−0.184, 0.131]	−0.351	−4.490^***^	[−0.504, −0.197]
Age	−0.040	−0.934	[−0.125, 0.044]	0.063	1.487	[−0.020, 0.147]	−0.023	−0.560	[−0.105, 0.058]
Childhood emotional trauma	0.185	4.565^***^	[0.106, 0.265]	−0.288	−7.206^***^	[−0.367, −0.210]	0.103	2.542^*^	[0.024, 0.183]
Left-behind experience	−0.058	−0.712	[−0.222, 0.105]	0.155	1.928	[−0.003, 0.313]	−0.016	−0.204	[−0.170, 0.138]
Childhood emotional trauma × left-behind experience	0.034	0.418	[−0.124, 0.192]	0.034	0.418	[−0.124, 0.192]	−0.104	1.266	[−0.057, 0.264]
Psychological resilience							−0.281	−6.905^***^	[−0.361, −0.201]
Psychological resilience × left-behind experience							0.174	2.123^*^	[0.013, 0.334]
*R^2^*		0.068			0.098			0.148	
*F*		6.694^***^			12.348^***^			14.084^***^	

*N* = 577. The bootstrap method was used for calculating the correlation coefficients. Gender is a dummy variable coded 1 for girls and 0 for boys, and the mean represents the proportion of girls. ^*^p < 0.05, ^**^p < 0.01, ^***^p < 0.00.

**Figure 2:**
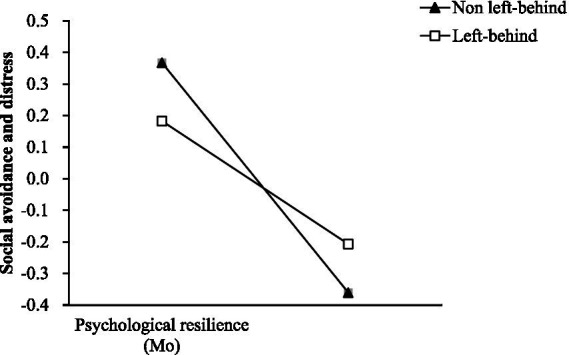
Simple slope diagram.

In order to understand the essence of moderation, a simple slope test was conducted (Hayes, 2013). The results (Figure 2) show that for junior high school students who have no experience of being left behind, psychological resilience has a significant negative predictive effect on social avoidance and distress (*β_simple_* = 0.111, *p* < 0.001). For junior high school students with left-behind experiences, psychological resilience also has a significant negative predictive effect on social avoidance and distress, albeit with a relatively smaller predictive effect (*β_simple_* = 0.529, *p* < 0.001). This suggests that, compared to those with left-behind experience, the moderating effect is more pronounced among junior high school students who have not experienced being left behind.

## 4 Discussion

Based on the dual-risk model of qualitative stress theory (Belsky and Pluess, 2009), this study explored the underlying mechanisms leading to social avoidance and distress among Chinese junior high school students. The results of this study show that childhood emotional trauma has a significant positive effect on social avoidance and distress. The higher the level of childhood emotional trauma, the more serious the social avoidance and distress become. This is consistent with previous research findings that middle school students who experienced emotional trauma in childhood have more social problems (Amanda and Richard, 2013). This result supports the dual-risk model of qualitative stress theory, which states that vulnerable individuals are prone to developmental problems (Belsky and Pluess, 2009). Additionally, this study supports the personality formation theory that negative attitudes of family caregivers toward children can lead to individuals developing strong anxiety about the outside world (Horney, 1994). The continued accumulation of anxiety increases the risk of psychological and behavioral problems in junior high school students, leading to social avoidance and distress.

### 4.1 The mediating role of psychological resilience

The results of the mediating effect analysis reveal that emotional trauma in childhood directly impacts social avoidance and distress among middle school students. Additionally, it indirectly influences these outcomes through psychological resilience. This underscores the crucial role of psychological resilience in safeguarding the mental health of individuals who have experienced childhood emotional trauma. According to the process model of psychological resilience proposed by Richardson (2002), individuals with strong psychological resilience excel at self-adjusting and adapting to their environment. Consequently, when confronted with negative events and stressful situations, junior high school students possessing high psychological resilience are able to leverage both internal and external resources effectively. They actively adjust their mindset and successfully navigate crises and interpersonal difficulties, thereby reducing social anxiety, fostering harmonious relationships, and enhancing their collective adaptation and growth.

The causative model of social anxiety suggests that negative life experiences, such as emotional trauma in childhood, can contribute to the occurrence of social problems (Nanda et al., 2016). In other words, emotional trauma in childhood is a predisposing factor for social issues in adolescents. However, there are still individuals who experience interpersonal anxiety but do not necessarily suffer from significant social problems. As a positive psychological quality, psychological resilience serves as an important protective factor for individuals susceptible to interpersonal anxiety related to social anxiety (Zeng et al., 2017). Social anxiety theory also points out that a poor cognitive assessment of social threat cues lies at the core of anxiety symptoms (Robert, 1990). When faced with stressful environments, individuals with low levels of psychological resilience are prone to negative perceptions of events, making it difficult for them to adapt, tolerate, and regulate their negative emotions. This, in turn, leads to a fear of coping with stimuli, thereby increasing the likelihood of social anxiety and avoidance.

### 4.2 The moderating effect of left-behind experience

Compared with previous studies focusing on the influence of childhood trauma on social avoidance and distress, this study constructed a moderated mediating model to investigate the moderating role of left-behind experience on the mediating path between childhood emotional trauma and social avoidance and distress among junior high school students. The results revealed that the left-behind experience functioned as a moderator in the second half of the mediation path. Specifically, when compared to the non-left-behind experience group, left-behind experience amplified the impact of childhood emotional trauma on social avoidance and distress among junior high school students. This is a novel finding from this study. On the one hand, it is possible that the left-behind experience results in parents being unable to accompany their children for extended periods, leading to insufficient care for their lives and emotions. This, in turn, may contribute to a decrease in the level of psychological resilience among junior high school students. On the other hand, parental companionship can facilitate individuals in learning positive social experiences from their parents, thereby enhancing their psychological resilience, enabling them to face life’s difficulties with positive attitudes and behaviors, and ultimately reducing the occurrence of social problems among junior high school students.

## 5 Conclusion

The current research holds crucial theoretical and practical contributions. Firstly, this study explored the impact of childhood emotional trauma on social avoidance and distress among junior high school students, further clarifying the mechanism of psychological resilience and left-behind experience, and enriching the relevant theoretical research on childhood trauma. Secondly, this study found that psychological resilience plays a pivotal role in the relationship between emotional trauma, social avoidance, and distress during childhood. This indicates that a warm, safe, and positive family atmosphere, as an important environment for individual growth, can assist middle school students in developing a positive social style. Concurrently, enhancing adolescents’ psychological resilience in secondary school education can help mitigate the impact of risk factors such as childhood emotional trauma on their social activities, thereby having significant practical implications for improving the mental health of junior high school students. Thirdly, this study examined the moderating role of left-behind experience in the mediating path between emotional trauma, social avoidance, and distress in childhood, confirming its significant impact on the mental health and social problems of junior high school students. This suggests that we must place greater emphasis on the growth and education of left-behind children. Society and families should strive to take various measures to minimize the number of left-behind children. Simultaneously, it is crucial to arrange for appropriate care and upbringing of left-behind children, maintaining regular contact or frequent visits to minimize the impact of the left-behind experience.

In summary, this study takes Chinese junior high school students as the research subjects, which is of great significance for understanding the mechanisms of emotional trauma, social avoidance, and distress in childhood. First, childhood emotional trauma can significantly predict the levels of social avoidance and distress among junior high school students. Second, psychological resilience plays a mediating role in the relationship between emotional trauma and social avoidance and distress in childhood. Improving the level of psychological resilience during the educational process can significantly reduce the levels of social avoidance and distress among junior high school students. Third, left-behind experience plays a moderating role in the second half of the mediation path between emotional trauma and social avoidance and distress in childhood. Compared with junior high school students without left-behind experience, those with left-behind experience tend to have a more significant impact of psychological resilience on social avoidance and distress.

When interpreting the findings, several limitations need to be taken into account. Firstly, this study utilizes cross-sectional data, which precludes the inference of causal relationships between variables. Therefore, it is necessary to employ longitudinal designs in future research to obtain stronger empirical evidence of causality. Secondly, although this study focuses on the relationship between childhood emotional trauma and social avoidance and distress, it has not examined whether individual emotional trauma actually occurs after separation from one or both parents. Finally, this study combined the two dimensions of emotional abuse and emotional neglect into a single composite score without explicitly testing their unidimensional structure. Future research should employ confirmatory factor analysis (CFA) to validate the unidimensionality of this construct.

## Data Availability

The datasets presented in this study can be found in online repositories. The names of the repository/repositories and accession number(s) can be found in the article/Supplementary material.
