# Supplementary material for: Childhood emotional trauma and social avoidance and distress in adolescents: psychological resilience as mediator and left-behind experience as moderator
Source: Front Psychol. 2025 Jul 14;16:1578809. doi: 10.3389/fpsyg.2025.1578809 (PMC12301316; doi:10.3389/fpsyg.2025.1578809)
Supplement: Supplementary file 1 [file Data_Sheet_1.pdf]

[1] C : \ U s e r s \ L e n o v o \ D e s k t o p \ \ \ \ . s a v

Run MATRIX procedure:

************** PROCESS Procedure for SPSS Version 3.2 ***************

Written by Andrew F. Hayes, Ph.D. www.afhayes.com
Documentation available in Hayes (2018). www.guilford.com/p/hayes3

****
Model : 59
Y : ZD
X : ZAQG
M : ZFF
W : _1

Covariates:
Sex_1 Age_1

Sample
Size: 577

****
OUTCOME VARIABLE:
ZFF

Model Summary

R	R-sq	MSE	F	df1	df2	p
.3124	.0976	.9103	12.3475	5.0000	571.0000	.0000

Model

	coeff	se	t	p	LLCI	ULCI
constant	-.8983	.6117	-1.4685	.1425	-2.0998	.3032
ZAQG	-.2880	.0400	-7.2055	.0000	-.3665	-.2095
_1	.1548	.0803	1.9281	.0543	-.0029	.3125
Int_1	.0336	.0804	.4183	.6759	-.1243	.1916
Sex_1	-.0266	.0802	-.3311	.7407	-.1841	.1310
Age_1	.0632	.0425	1.4870	.1376	-.0203	.1466

Product terms key:
Int_1 : ZAQG x _1

Covariance matrix of regression parameter estimates:

	constant	ZAQG	_1	Int_1	Sex_1	Age_1
constant	.3742	-.0003	-.0006	-.0012	.0025	-.0259
ZAQG	-.0003	.0016	.0003	.0001	.0000	.0000
_1	-.0006	.0003	.0064	.0000	.0001	.0000
Int_1	-.0012	.0001	.0000	.0065	.0003	.0001
Sex_1	.0025	.0000	.0001	.0003	.0064	-.0004

```
Age_1        -.0259      .0000      .0000      .0001     -.0004      .0018

Test(s) of highest order unconditional interaction(s):
       R2-chng          F        df1        df2          p
X*W      .0003      .1749     1.0000   571.0000      .6759
----------
    Focal predict: ZAQG     (X)
           Mod var: _1  (W)

Data for visualizing the conditional effect of the focal predictor:
Paste text below into a SPSS syntax window and execute to produce plot.

DATA LIST FREE/
   ZAQG        _1    ZFF         .
BEGIN DATA.
    -1.0000     -.4773      .2319
      .0000     -.4773     -.0722
     1.0000     -.4773     -.3763
    -1.0000      .0000      .2897
      .0000      .0000      .0017
     1.0000      .0000     -.2864
    -1.0000      .4977      .3500
      .0000      .4977      .0787
     1.0000      .4977     -.1926
END DATA.
GRAPH/SCATTERPLOT=
 ZAQG     WITH     ZFF      BY        _1 .

**************************************************************************
OUTCOME VARIABLE:
 ZD

Model Summary
          R       R-sq        MSE          F        df1        df2
  p
      .3843      .1477      .8628    14.0844     7.0000   569.0000      .0
000

Model
              coeff         se          t          p       LLCI       ULCI
constant      .4947      .5967      .8290      .4074     -.6773     1.6667
ZAQG          .1034      .0406     2.5428      .0113      .0235      .1832
ZFF          -.2813      .0407    -6.9048      .0000     -.3614     -.2013
_1     -.0160      .0784     -.2035      .8388     -.1700      .1381
Int_1         .1035      .0817     1.2660      .2060     -.0571      .2641
Int_2         .1737      .0818     2.1231      .0342      .0130      .3344
Sex_1        -.3508      .0781    -4.4904      .0000     -.5042     -.1973
Age_1        -.0232      .0414     -.5599      .5758     -.1046      .0582

Product terms key:
 Int_1    :        ZAQG     x        _1
 Int_2    :        ZFF      x        _1
```

```
Covariance matrix of regression parameter estimates:
             constant        ZAQG         ZFF    _1       Int_1       Int_2
   Sex_1      Age_1
constant      .3561       .0001       .0015      -.0008      -.0010       .0006
      .0024     -.0246
ZAQG          .0001       .0017       .0005       .0002       .0001       .0000
      .0000      .0000
ZFF           .0015       .0005       .0017      -.0003       .0000       .0000
      .0000     -.0001
_1     -.0008       .0002     -.0003       .0062       .0000      -.0001
   .0001      .0001
Int_1        -.0010       .0001       .0000       .0000       .0067       .0019
      .0002      .0001
Int_2         .0006       .0000       .0000      -.0001       .0019       .0067
     -.0001     -.0001
Sex_1         .0024       .0000       .0000       .0001       .0002      -.0001
      .0061     -.0004
Age_1        -.0246       .0000      -.0001       .0001       .0001      -.0001
     -.0004      .0017

Test(s) of highest order unconditional interaction(s):
       R2-chng          F        df1        df2          p
X*W      .0024     1.6027     1.0000   569.0000      .2060
M*W      .0068     4.5074     1.0000   569.0000      .0342
----------
    Focal predict: ZAQG     (X)
           Mod var: _1  (W)

Data for visualizing the conditional effect of the focal predictor:
Paste text below into a SPSS syntax window and execute to produce plot.

DATA LIST FREE/
   ZAQG        _1    ZD          .
BEGIN DATA.
    -1.0000     -.4773     -.0503
      .0000     -.4773      .0037
     1.0000     -.4773      .0576
    -1.0000      .0000     -.1073
      .0000      .0000     -.0040
     1.0000      .0000      .0994
    -1.0000      .4977     -.1668
      .0000      .4977     -.0119
     1.0000      .4977      .1430
END DATA.
GRAPH/SCATTERPLOT=
 ZAQG     WITH     ZD       BY       _1 .
----------
    Focal predict: ZFF      (M)
           Mod var: _1  (W)

Conditional effects of the focal predictor at values of the moderator(s):

   _1     Effect          se          t          p        LLCI        ULCI
```

-.4773	-.3642	.0561	-6.4932	.0000	-.4744	-.2541
.0000	-.2813	.0407	-6.9048	.0000	-.3614	-.2013
.4977	-.1949	.0579	-3.3632	.0008	-.3087	-.0811

There are no statistical significance transition points within the observed range of the moderator found using the Johnson-Neyman method.

Conditional effect of focal predictor at values of the moderator:

_1	Effect	se	t	p	LLCI	ULCI
-.4773	-.3642	.0561	-6.4932	.0000	-.4744	-.2541
-.4273	-.3555	.0534	-6.6625	.0000	-.4604	-.2507
-.3773	-.3469	.0508	-6.8254	.0000	-.4467	-.2470
-.3273	-.3382	.0485	-6.9748	.0000	-.4334	-.2429
-.2773	-.3295	.0464	-7.1019	.0000	-.4206	-.2384
-.2273	-.3208	.0446	-7.1957	.0000	-.4084	-.2332
-.1773	-.3121	.0431	-7.2445	.0000	-.3968	-.2275
-.1273	-.3034	.0419	-7.2364	.0000	-.3858	-.2211
-.0773	-.2948	.0412	-7.1618	.0000	-.3756	-.2139
-.0273	-.2861	.0408	-7.0153	.0000	-.3662	-.2060
.0227	-.2774	.0408	-6.7973	.0000	-.3575	-.1972
.0727	-.2687	.0412	-6.5147	.0000	-.3497	-.1877
.1227	-.2600	.0421	-6.1794	.0000	-.3427	-.1774
.1727	-.2513	.0433	-5.8068	.0000	-.3364	-.1663
.2227	-.2427	.0448	-5.4127	.0000	-.3307	-.1546
.2727	-.2340	.0467	-5.0117	.0000	-.3257	-.1423
.3227	-.2253	.0488	-4.6153	.0000	-.3212	-.1294
.3727	-.2166	.0512	-4.2322	.0000	-.3171	-.1161
.4227	-.2079	.0538	-3.8680	.0001	-.3135	-.1023
.4727	-.1992	.0565	-3.5258	.0005	-.3102	-.0882

```
      .5227     -.1906      .0594    -3.2071      .0014     -.3073     -.0
739

Data for visualizing the conditional effect of the focal predictor:
Paste text below into a SPSS syntax window and execute to produce plot.

DATA LIST FREE/
   ZFF        _1    ZD          .
BEGIN DATA.
    -1.0000     -.4773      .3679
      .0000     -.4773      .0037
     1.0000     -.4773     -.3606
    -1.0000      .0000      .2774
      .0000      .0000     -.0040
     1.0000      .0000     -.2853
    -1.0000      .4977      .1830
      .0000      .4977     -.0119
     1.0000      .4977     -.2068
END DATA.
GRAPH/SCATTERPLOT=
 ZFF      WITH     ZD       BY        _1 .

****************** DIRECT AND INDIRECT EFFECTS OF X ON Y *****************

Conditional direct effect(s) of X on Y:
   _1     Effect         se          t          p       LLCI       ULCI
     -.4773      .0540      .0558      .9664      .3343     -.0557      .1
636
      .0000      .1034      .0406     2.5428      .0113      .0235      .1
832
      .4977      .1549      .0580     2.6690      .0078      .0409      .2
688

Conditional indirect effects of X on Y:

INDIRECT EFFECT:
 ZAQG        ->    ZFF         ->    ZD

   _1     Effect     BootSE   BootLLCI   BootULCI
     -.4773      .1108      .0309      .0584      .1811
      .0000      .0810      .0191      .0475      .1234
      .4977      .0529      .0231      .0165      .1072

 Pairwise contrasts between conditional indirect effects (Effect1 minus Ef
fect2)
    Effect1    Effect2   Contrast     BootSE   BootLLCI   BootULCI
      .0810      .1108     -.0297      .0223     -.0786      .0104
      .0529      .1108     -.0579      .0384     -.1365      .0166
      .0529      .0810     -.0282      .0165     -.0600      .0061
---

**************************************************************************
Bootstrap estimates were saved to a file
```

```
Map of column names to model coefficients:
          Conseqnt Antecdnt
 COL1     ZFF      constant
 COL2     ZFF      ZAQG
 COL3     ZFF      _1
 COL4     ZFF      Int_1
 COL5     ZFF      Sex_1
 COL6     ZFF      Age_1
 COL7     ZD       constant
 COL8     ZD       ZAQG
 COL9     ZD       ZFF
 COL10    ZD       _1
 COL11    ZD       Int_1
 COL12    ZD       Int_2
 COL13    ZD       Sex_1
 COL14    ZD       Age_1

*********** BOOTSTRAP RESULTS FOR REGRESSION MODEL PARAMETERS ************

OUTCOME VARIABLE:
 ZFF

              Coeff   BootMean     BootSE   BootLLCI   BootULCI
constant     -.8983     -.9082      .6218    -2.1111      .3011
ZAQG         -.2880     -.2910      .0445     -.3811     -.2055
_1      .1548      .1544      .0802     -.0064      .3092
Int_1         .0336      .0323      .0900     -.1471      .2104
Sex_1        -.0266     -.0293      .0803     -.1829      .1278
Age_1         .0632      .0638      .0431     -.0199      .1471

----------

OUTCOME VARIABLE:
 ZD

              Coeff   BootMean     BootSE   BootLLCI   BootULCI
constant      .4947      .4936      .6100     -.6913     1.6568
ZAQG          .1034      .0994      .0529     -.0057      .2020
ZFF          -.2813     -.2827      .0412     -.3642     -.2006
_1     -.0160     -.0178      .0783     -.1735      .1310
Int_1         .1035      .0993      .1063     -.1063      .3062
Int_2         .1737      .1739      .0832      .0086      .3395
Sex_1        -.3508     -.3508      .0773     -.5017     -.2000
Age_1        -.0232     -.0232      .0424     -.1053      .0597

********************** ANALYSIS NOTES AND ERRORS ************************

Level of confidence for all confidence intervals in output:
  95.0000

Number of bootstrap samples for percentile bootstrap confidence intervals:
  5000
```

```
W values in conditional tables are the minimum, the mean, and 1 SD above t
he mean.

NOTE: One SD below the mean is below the minimum observed in the data for
W,
      so the minimum measurement on W is used for conditioning instead.

NOTE: The following variables were mean centered prior to analysis:
           _1 ZAQG      ZFF

NOTE: Variables names longer than eight characters can produce incorrect o
utput.
      Shorter variable names are recommended.

------ END MATRIX -----
```
