# Supplementary material for: Childhood emotional trauma and social avoidance and distress in adolescents: psychological resilience as mediator and left-behind experience as moderator
Source: Front Psychol. 2025 Jul 14;16:1578809. doi: 10.3389/fpsyg.2025.1578809 (PMC12301316; doi:10.3389/fpsyg.2025.1578809)
Supplement: Supplementary file 2 [file Data_Sheet_2.pdf]

Bootstrap

[1] C:\Users\Lenovo\Desktop\\\\.sav

Bootstrap

	5000<br>95.0%
