# Supplementary material for: Childhood emotional trauma and social avoidance and distress in adolescents: psychological resilience as mediator and left-behind experience as moderator
Source: Front Psychol. 2025 Jul 14;16:1578809. doi: 10.3389/fpsyg.2025.1578809 (PMC12301316; doi:10.3389/fpsyg.2025.1578809)
Supplement: Supplementary file 3 [file Data_Sheet_3.pdf]

[1] C : \ U s e r s \ L e n o v o \ D e s k t o p \ \ \ \ . s a v

			N
AQG	16.2547	5.71209	577
FF	90.0120	13.17668	577
D	16.1848	6.15844	577
SMEAN(Sex)	.47	.499	577
SMEAN(Age)	14.45	.942	577

		AQG	FF	D	SMEAN(Sex)
AQG	P e a r s o n	1	-.297**	.189**	-.005
			.000	.000	.913
		18793.708	-12862.618	3833.714	-7.502
		32.628	-22.331	6.656	-.013
	N	577	577	577	577
FF	P e a r s o n	-.297**	1	-.313**	-.007
		.000		.000	.860
		-12862.618	100007.940	-14650.034	-27.855
		-22.331	173.625	-25.434	-.048
	N	577	577	577	577
D	P e a r s o n	.189**	-.313**	1	-.175**
		.000	.000		.000
		3833.714	-14650.034	21845.591	-310.741
		6.656	-25.434	37.926	-.539
	N	577	577	577	577
SMEAN(Sex)	P e a r s o n	-.005	-.007	-.175**	1
		.913	.860	.000	
		-7.502	-27.855	-310.741	143.591
		-.013	-.048	-.539	.249
	N	577	577	577	577
SMEAN(Age)	P e a r s o n	-.010	.059	-.059	.112**
		.805	.154	.159	.007
		-31.865	425.367	-196.087	30.435
		-.055	.738	-.340	.053
	N	577	577	577	577

		SMEAN(Age)
AQG	P e a r s o n	-.010
		.805
		-31.865
		-.055
	N	577
FF	P e a r s o n	.059
		.154
		425.367
		.738
	N	577
D	P e a r s o n	-.059
		.159
		-196.087
		-.340
	N	577
SMEAN(Sex)	P e a r s o n	.112**
		.007
		30.435
		.053
	N	577
SMEAN(Age)	P e a r s o n	1
		511.614
		.888
	N	577

**. 在 .01 水平（双侧）上显著相关。
